# Serpin family A member 1 is an oncogene in glioma and its translation is enhanced by NAD(P)H quinone dehydrogenase 1 through RNA-binding activity

**DOI:** 10.1515/med-2022-0572

**Published:** 2022-10-20

**Authors:** Wenjun Liu, Min Du, Hongping Wan, Hao Yang, Xiaorong Deng, Yu Chen, Qian Zhang

**Affiliations:** Department of Neurology, Hubei No. 3 People’s Hospital of Jianghan University, Wuhan, Hubei, China; Department of Neurology, Hubei No. 3 People’s Hospital of Jianghan University, No. 26 Zhongshan Avenue, Qiaokou District, Wuhan, Hubei, China

**Keywords:** glioma, *SERPINA1*, *NQO1*, RNA-binding protein, miR-1321

## Abstract

Serpin family A member 1 (*SERPINA1*) is expressed abundantly in gliomas and can predict unfavorable prognosis of patients with glioma. Studies have shown that nicotinamide adenine dinucleotide phosphate quinone dehydrogenase 1 (*NQO1*) can promote the proliferation of glioblastoma multiforme cells and enhance the expression of *SERPINA1*, but its effects on glioma cells remain unknown. In this study, we explored the functions of *SERPINA1* in glioma tumorigenesis *in vitro* and then investigated whether *NQO1* affects the protein expression of *SERPINA1* and its mRNA level. The results showed that the translation of *SERPINA1* was suppressed while its mRNA level had no significant changes under the condition of *NQO1* silencing. Luciferase reporter assay and biotin pull-down assay further indicated that *NQO1* bond with *SERPINA1* 3′ untranslated region. miR-1321 was also identified to target *SERPINA1*, repressing its mRNA and protein levels*. SERPINA1* and *NQO1* promoted glioma cell proliferation and suppressed cell apoptosis. Moreover, *SERPINA1* rescued the effects of sh-NQO1 in glioma cell malignant phenotypes. In conclusion, our findings showed that oncogene *NQO1* and antioncogene miR-1321 bind to oncogene *SERPINA1* to affect proliferation and apoptosis of glioma cells, which can bring new solution of antitumor treatments for glioma in the future.

## 1 Introduction

Glioma, known as an aggressive malignant cancer in brain, causes nearly 80% of malignant brain cancers in the world with a high rate of relapse [1,2,3]. It is characterized by infiltrative growth of malignant glioma cells into the surrounding brain parenchyma, which makes it hard for operative treatment. Although treatment for neuronal oncology has been improved rapidly, the prognosis of glioma is unsatisfactory [4]. Novel treatment strategies for malignant gliomas should focus on improving tumor debulking and enhancing tumor cell killing [5]. Glioblastoma multiforme (GBM) is a highly immunosuppressive tumor, and immunotherapy can achieve long-lasting tumor remission by manipulating the immune system [6]. However, immunotherapy may cause over-inflammation, and its efficacy is not high [7]. Therefore, it is important to develop new strategies for the treatment of glioma.

Serpin family A member 1 (*SERPINA1*), identified in 1981 [8], serves as a serine protease inhibitor gene. It was reported that in patients with carcinomas, *SERPINA1* enhances the invasive and metastatic abilities of carcinomas in gastric cancer, colorectal carcinoma, and lung cancer [9,10,11,12,13] and indicates poor prognosis of patients with colorectal cancer [14]. The expression of *SERPINA1* was found in the spindle cells and pleomorphic cells from the sarcomatous area of GBM [15,16]. An immunostaining result demonstrates the expression of *SERPINA1* in gliomas [17]. In this study, we used short hairpin RNAs (shRNAs) to determine the effects of *SERPINA1* on the proliferation and apoptosis of glioma cells.

Nicotinamide adenine dinucleotide phosphate quinone oxidoreductase (*NQO1*), one of the major cytosolic quinone reductases, induces a two-electron reduction of quinones substrate to hydroquinones. Previous studies showed that *NQO1* increases glioma cell proliferation [18,19]. Meanwhile, new evidence suggested that the expression of *SERPINA1* is upregulated by *NQO1* through RNA-binding activity [20]. However, the property of the binding of *NQO1* to *SERPINA1* in glioma cells remains unknown.

We made a hypothesis that *NQO1* binds to *SERPINA1* to play an oncogenic role in glioma cells. A noncoding RNA miR-1321 targeting *SERPINA1* was subsequently investigated. Influences of *NQO1*, *SERPINA1*, and miR-1321 on the proliferation and apoptosis of glioma cells were assessed.

## 2 Methods and materials

### 2.1 Bioinformatics analysis

Interactive Analysis of Gene Expression Profiles, abbreviated as GEPIA (http://gepia.cancer-pku.cn/), is a web server to analyze the expression of *SERPINA1* in glioma and normal tissues from the TCGA and GTEx projects. Association of *NQO1* and *SERPINA1* with the prognosis of glioma patients as well as the expression correlation between *NQO1* and *SERPINA1* in glioma tissues were also obtained from GEPIA.

### 2.2 Cell culture

The normal human astrocyte (NHA) (Cloneticss Astrocyte Cell Systems, Cambrex BioScience, Wokingham, UK) and four glioma cell lines (U251, T98G, LN-229, and A172) (Shanghai Institute of Cell Biology, Chinese Academy of Sciences, Shanghai, China) were used. The GBM cells were first stored in 5% CO_2_ at 37°C in a humidified chamber and then cultured with 10% fetal bovine serum [21]. DMEM supplemented with 100 units/mL penicillin, 100 μg/mL streptomycin, and 2 mM Glutamax (Invitrogen) was used to culture cells under standard cell culture conditions. Primary glioma cells were purchased from PriCells (HUM-TUM-0018, Wuhan, China) and cultured in special basal medium for primary glial cells (MED-0014, PriCells) supplemented with PBS and antibiotics.

### 2.3 Cell transfection

A *SERPINA1* complementary DNA (cDNA) was cloned into the pcDNA3.1 vector (Genechem, Shanghai, China) to produce the *SERPINA1* overexpressing vector. The shRNAs used for targeting *SERPINA1* and *NQO1* were synthesized by Genechem. MiR-1321 inhibitor for silencing miR-1321 expression and miR-1321 mimics for enhancing miR-1321 expression were synthesized by GenePharma (Shanghai, China). Lipofectamine 2000 (Invitrogen, Carlsbad, CA, USA) was used for the transfection of abovementioned oligonucleotides or vectors into T98G and A172 cells for 24 h.

### 2.4 Colony formation assay

Cells were plated in six-well plates (1,000 cells per well) for cell culture for 2 weeks. Next, 75% alcohol and 1% crystal violet were used to fix and stain the colonies.

### 2.5 Flow cytometry

The evaluation of apoptosis was taken using flow cytometry with Annexin V (PE)/7AAD (BD Biosciences, San, Jose, CA, USA) double staining. Annexin V/7AAD solution was used to incubate cells for 15 min at room temperature in the dark after transfection. A FACSCanto™ system (BD Biosciences) was used to analyze the samples. Annexin V and 7AAD positive cells were late apoptotic cells. Annexin V positive and 7AAD-negative cells were early apoptotic cells. Apoptotic rate was calculated as the percentage of early apoptotic cells + percentage of late apoptotic cells.

### 2.6 RNA isolation and RT-qPCR analysis

TRIzol was used to isolate RNA from cell extracts according to manufacturer’s procedures. An iScript™ Advanced cDNA Synthesis Kit for RT-qPCR (Bio-Rad) was used for reverse transcription. iTaq™ Universal SYBR® Green Supermix (Bio-Rad) was used for real-time quantitative (q)PCR analysis. *GAPDH* was used as a reference gene. The primer used for amplification are listed as the following:

*SERPINA1*: F: 5′-ATCATAGGCACCTTCACGG-3′; R: 5′-TCTTTAAAGGCAAATGGGAGAG-3′.

*NQO1*: F: 5′-ACATCACAGGTAAACTGAAGG-3′; R: 5′-TCAGATGGCCTTCTTTATAAGC-3′.

*GAPDH*: F: 5′-TCAAGATCATCAGCAATGCC-3′; R: 5′-CGATACCAAAGTTGTCATGGA-3′.

### 2.7 Luciferase reporter assay

The psiCHECK2 Luciferase reporter vector (Promega, Madison, WI, USA) was used to load the 3′-UTR, coding regions, and 5′-UTR of *SERPINA1*. A172 and T98G cells were seeded into 24-well plates. Then, sh-DQO1 plasmid and luciferase reporter vectors were co-transfected into the cells for 24 h using the Effectene transfection reagent (Qiagen, Hilden, Germany). After that, a Dual-Luciferase assay system (Promega, Madison, WI, USA) was used to measure the Firefly luciferase (FL) activity and Renilla luciferase (RL) activity. The results were shown as the normalized value of FL to RL.

### 2.8 Immunofluorescence staining

Four percent paraformaldehyde was used to fix cells for 30 min. Five percent blocking solution was used for permeabilization. An anti-SERPINA1 antibody (ab207303, 1:1,000; Abcam, Cambridge, UK) was used to incubate cells overnight at 4°C followed by washing with phosphate-buffered saline. A goat anti-rabbit IgG Alexa Fluor® 647 antibody (ab150075, 1:1,000; Abcam) was used as second antibody and used to incubate the membrane for 2 h. 4′,6-Diamidino-2-phenylindole (DAPI) was used to counterstain cell nuclei. At last, a fluorescence microscope (IX71, Olympus Corporation, Tokyo, Japan) was used to capture images.

### 2.9 Western blot

RIPA buffer was used to prepare whole-cell lysates. Five micrograms of proteins were isolated with 4–15% Criterion™ TGX™ precast gels (Bio-Rad) by electrophoresis and transferred to nitrocellulose membrane using Trans-Blot Turbo™ Transfer System (Bio-Rad). After that, primary antibodies against NQO1 (ab80588, 1:10,000; Abcam), *SERPINA1* (ab207303, 1:5,000; Abcam), and GAPDH (ab8245, 1:1,000, Abcam) were added for incubation overnight at 4°C. Next, appropriate HRP-labeled secondary antibody IgG (ab7090, 1:1,000; Abcam) was added. Enhanced chemiluminescence (Amersham) was used to detect immunocomplexes. At last, quantitative densitometry of the protein bands was performed with the ImageJ software (National Institutes of Health).

### 2.10 Biotin pull-down assay

Primers containing the T7 RNA polymerase promoter sequence were used as templates for PCR. *In vitro* transcription was performed with a MegaScript T7 kit (Ambion). Purification was taken using Nuc-Away Spin Columns (Applied Biosystems). Cytoplasmic lysates from A172 and T98G cells (150 μg lysate, 1 μg biotinylated RNA) were incubated with biotinylated transcripts for 30 min at room temperature. Streptavidin-coated magnetic Dynabeads (Dynal) were used to isolate complexes. NQO1 protein was detected by western blot. The primer used to prepare biotinylated *SERPINA1* 3′UTR is listed as follows: forward, 5′-AGTAATACGACTCACTATAGGGCCCAGAACTGCCTGATCGTG-3′; reverse, 5′-GCCATTCCTGGTAGAGACGG-3′.

### 2.11 Statistical analysis

Statistical differences were determined by unpaired Student’s *t* test between two groups or one-way analysis of variance (ANOVA) among three or more groups. GraphPad Prism 6.0 was used to generate *p* values (*p* < 0.05 indicates significance). Error bar represents standard error of the mean (SEM).

## 3 Results

### 3.1 *SERPINA1* is Upregulated in Glioma

**Figure 1:**
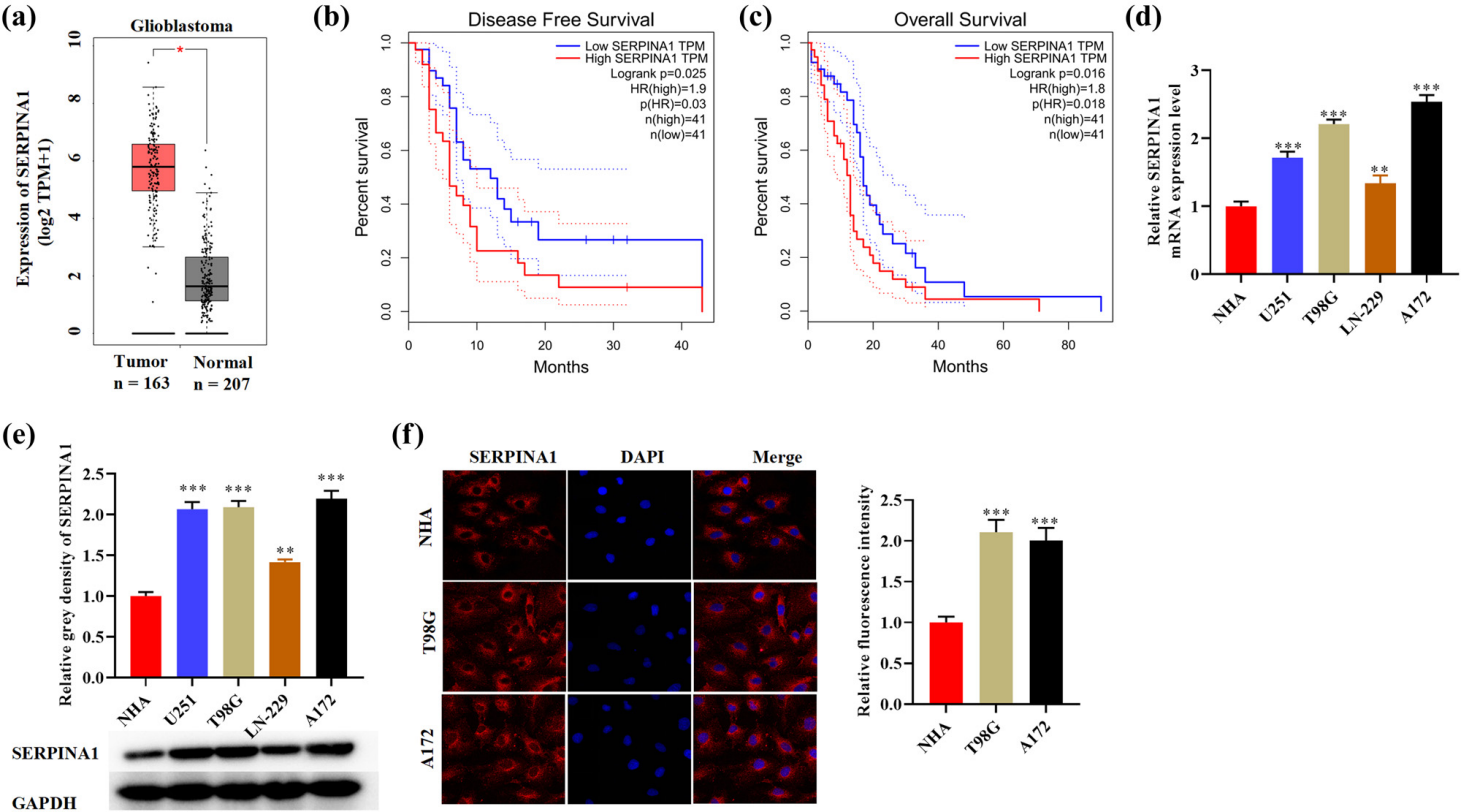
*SERPINA1* is upregulated in glioma. (a) Differential expression of *SERPINA1* in glioblastoma (box plot) (red: tumor tissue, gray: normal tissue). (b and c) Kaplan–Meier survival curves of disease-free survival and overall survival of patients who had gliomas with differential expression of *SERPINA1*. (d and e) qRT-PCR analysis of *SERPINA1* mRNA expression and western blotting analysis of *SERPINA1* protein in NHA, U251, T98G, LN-229, and A172 cell lines. One-way ANOVA was conducted. (f) Co-staining of *SERPINA1* (red) with DAPI (blue) in NHA, T98G, and A172 cell lines. One-way ANOVA was conducted. Data are expressed as means ± SEM (*p < 0.05; **p < 0.01; ***p < 0.001, *n* = 3).

Using the GEPIA dataset, we obtained *SERPINA1* expression in glioma tumors and normal tissues. The findings revealed that glioblastoma has higher *SERPINA1* expression level than normal tissue (*p* < 0.05) (Figure 1a). The survival analysis suggested that glioma patients with high level of *SERPINA1* expression had poor survival (Figure 1b and c). The expression of *SERPINA1* at the messenger RNA (mRNA) and protein levels was also measured in NHA and four glioma cell lines, which showed that the expression of *SERPINA1* was increased remarkably in glioma cells (Figure 1d and e). Higher expression of *SERPINA1* in T98G and A172 cells than NHA was confirmed by immunofluorescence staining, which also indicates the cytoplastic location of *SERPINA1* (Figure 1f). These results suggest that *SERPINA1* might indicate poor prognosis in gliomas.

### 3.2 *SERPINA1* affects the proliferation and apoptosis of glioma cells

**Figure 2:**
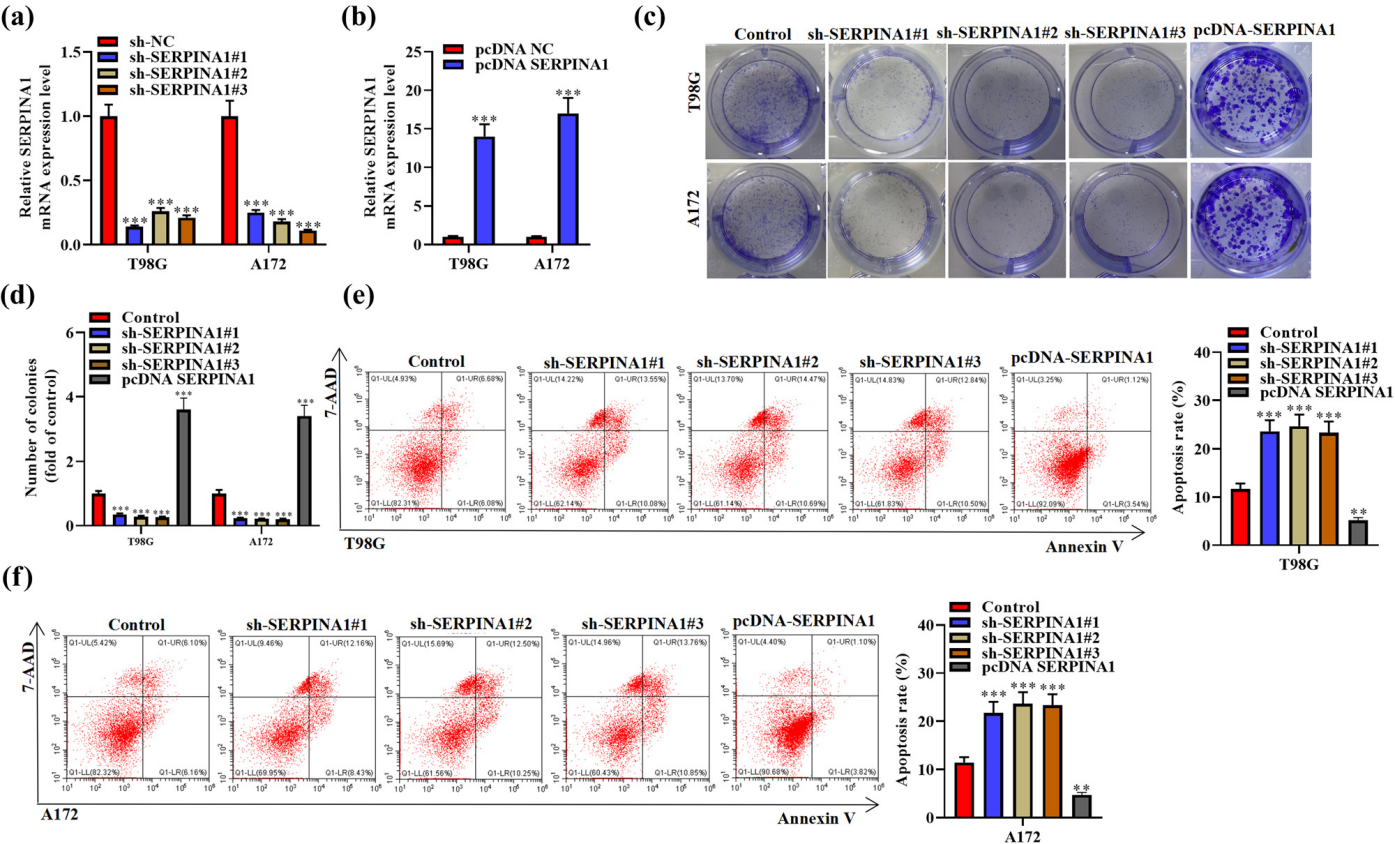
*SERPINA1* affects the proliferation and apoptosis of glioma cells. (a) Relative *SERPINA1* mRNA expression level in T98G and A172 cells transfected with sh-SERPINA1, determined by qRT-PCR assay. One-way ANOVA was conducted. (b) Relative *SERPINA1* mRNA expression level in T98G and A172 cells transfected with pcDNA *SERPINA1*, determined by qRT-PCR assay. Student’s *t* test was conducted. (c and d) Colony formation assay and its quantitative diagram. One-way ANOVA was conducted. (e) Annexin V/7AAD double staining assay and apoptosis rate in T98G. One-way ANOVA was conducted. (f) Annexin V/7AAD double staining assay and apoptosis rate in A172. One-way ANOVA was conducted. Data are expressed as means ± SEM (**p < 0.01; ***p < 0.001, *n* = 3).

The relative *SERPINA1* expression was reduced with transfection of sh-SERPINA1#1/2/3 (Figure 2a) and increased significantly with transfection of pcDNA *SERPINA1* (Figure 2b). Then, colony formation assay was taken to analyze the impact of *SERPINA1* silence on the proliferation of glioma cells. The proliferation was significantly suppressed by sh-SERPINA1 and promoted by pcDNA *SERPINA1* (Figure 2c and d). To explore whether sh-SERPINA1 inhibits cell proliferation by enhancing apoptosis, a flow cytometry assay (Annexin V/7AAD staining) was conducted. The results showed that in glioma cells, apoptosis rate was increased when *SERPINA1* was silenced (Figure 2e and f). These findings showed that *SERPINA1* promotes the proliferative ability of glioma cells and suppresses apoptosis.

### 3.3 *NQO1* enhanced *SERPINA1* mRNA translation through binding to the 3′UTR

**Figure 3:**
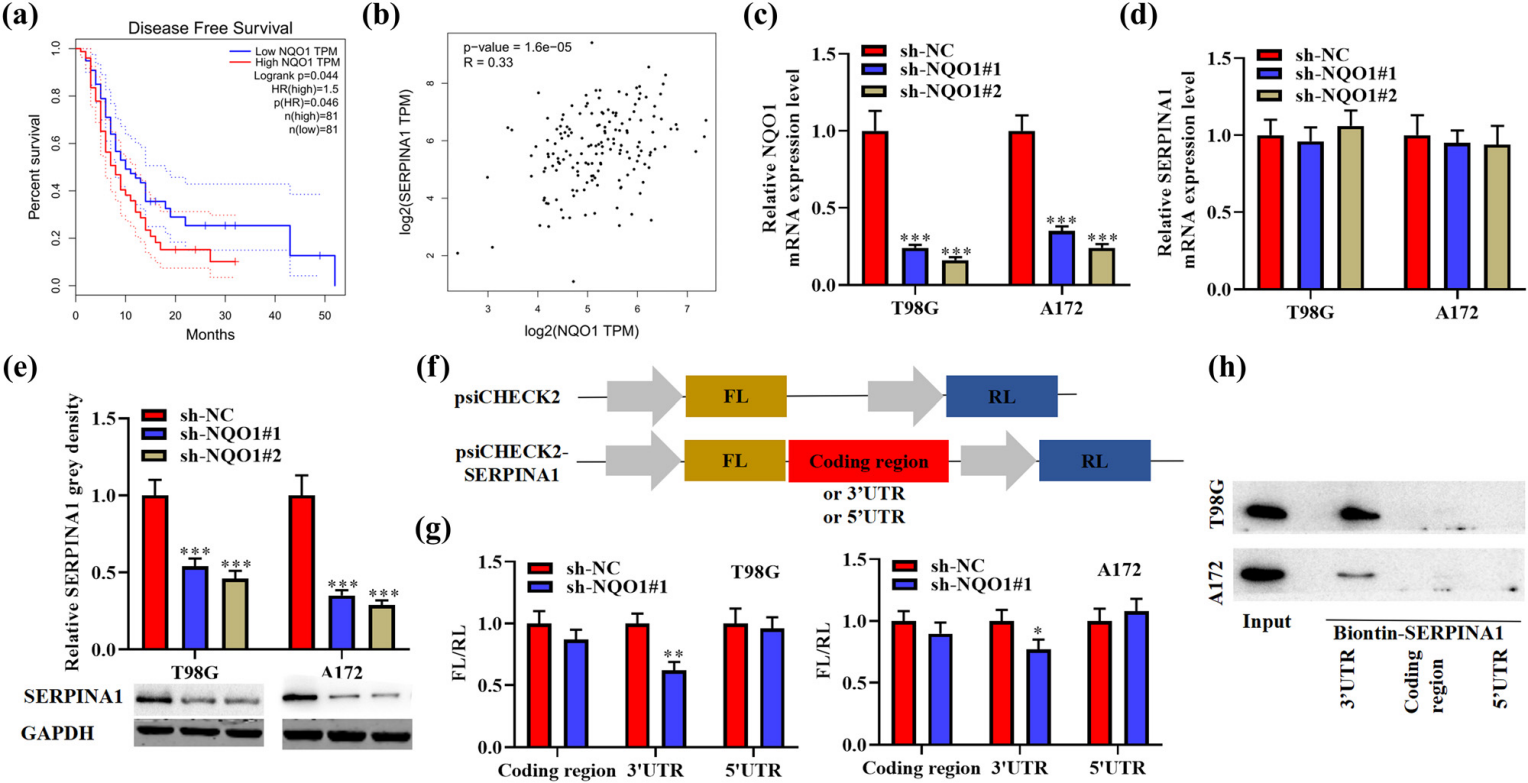
*NQO1* enhanced *SERPINA1* mRNA translation through binding to the 3′UTR. (a) Kaplan–Meier survival curves of disease-free survival of patients who had gliomas with differential expression of *NQO1*. (b) Correlation between *NQO1* and *SERPINA1* expression in glioma tissues. (c) Relative *NQO1* mRNA expression level in T98G and A172 cells transfected with sh-NQO1, determined by qRT-PCR assay. One-way ANOVA was conducted. (d) Relative *SERPINA1* mRNA expression level in T98G and A172 cells transfected with sh-NQO1, determined by qRT-PCR assay. One-way ANOVA was conducted. (e) Relative *SERPINA1* grey densities in A172 and T98G cells after transfection with sh-NQO1. One-way ANOVA was conducted. (f) 3′UTR, coding region, and 5′UTR of *SERPINA1* mRNA were subcloned downstream FL coding sequence, with RL expressed from the same construct as control. (g) The ratios of FL/RL activity in glioma cells. *NQO1* silencing inhibited FL/RL reporter activity in T98G and A172 cells. Student’s *t* test was conducted. (h) RNA pull-down assay followed by western blot revealed the existence of *NQO1* protein that is pulled down by biotin-labelled *SERPINA1* 3′UTR. Data are expressed as means ± SEM (*p < 0.05; **p < 0.01; ***p < 0.001, *n* = 3).

The survival analysis showed that high level of *NQO1* is associated with the low survival rates of glioma patients (Figure 3a). *SERPINA1* expression had positive correlation with *NQO1* expression in glioblastoma (Figure 3b). The relative mRNA level of *NQO1* was suppressed in T98G and A172 cells after transfection with sh-NQO1 (Figure 3c). Silence of *NQO1* reduced the protein expression of *SERPINA1* without causing significant changes in its mRNA level (Figure 3d–e). To explore *NQO1* binds to which region of *SERPINA1*, a luciferase reporter construct (psiCHECK2) bearing the RL coding region was used to load *SERPINA1* 3′UTR, coding region, and 5′UTR (Figure 3f). The silence of *NQO1* obviously reduced the activity of psiCHECK2-SERPINA1-3′UTR and had no significant effects on plasmids containing *SERPINA1* coding region and 5′UTR, indicating that *NQO1* directly binds with *SERPINA1* 3′UTR (Figure 3g). Binding assays using biotinylated transcripts indicated that in both A172 and T98G lysates, *NQO1* has significant interaction with the 3′UTR of *SERPINA1* mRNA (Figure 3h).

### 3.4 NQO1 boosts the proliferation of glioma cells by reducing apoptosis

**Figure 4:**
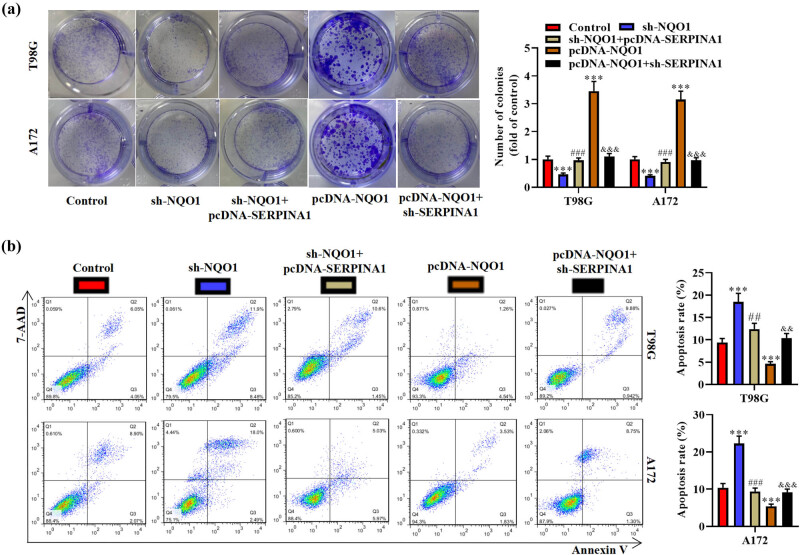
*NQO1* boosts the proliferation of glioma cells by reducing apoptosis. (a) Colony formation assay and quantitative analysis of T98G and A172 cells transfected with different plasmids (control, sh-NQO1, sh-NQO1 + pcDNA-SERPINA1, pcDNA-NQO1, pcDNA-NQO1 + sh-SERPINA1). One-way ANOVA was conducted. (b) Annexin V/7AAD double staining assay was used to show cell apoptosis rate in T98G and A172 cells. One-way ANOVA was conducted. Data are expressed as means ± SEM (***p < 0.001 vs control, ^##^ *p* < 0.01, ^###^ *p* < 0.001 vs sh-NQO1, ^&&^ *p* < 0.01, ^&&&^ *p* < 0.001 vs pcDNA-NQO1, *n* = 3).

The colony formation ability of glioma cells was suppressed by sh-NQO1 and enhanced by pcDNA-NQO1. pcDNA-SERPINA1 rescued the effects of sh-NQO1 on cell proliferation and sh-SERPINA1 had the same rescue function on pcDNA-NQO1 (Figure 4a). Furthermore, Annexin V/7AAD staining followed by flow cytometry analysis showed that pcDNA-SERPINA1 suppressed the apoptosis rate that is promoted by sh-NQO1, whereas sh-SERPINA1 restored the declined apoptosis rate that is induced by pcDNA-NQO1 (Figure 4b). These findings suggested that *NQO1* facilitates the proliferation of glioma cells by suppressing the apoptosis, which depends on the translation of *SERPINA1*.

### 3.5 NQO1 suppresses *SERPINA1* translation and *SERPINA1* rescues effects of sh-NQO1 on proliferation and apoptosis of primary glioma cells

**Figure 5:**
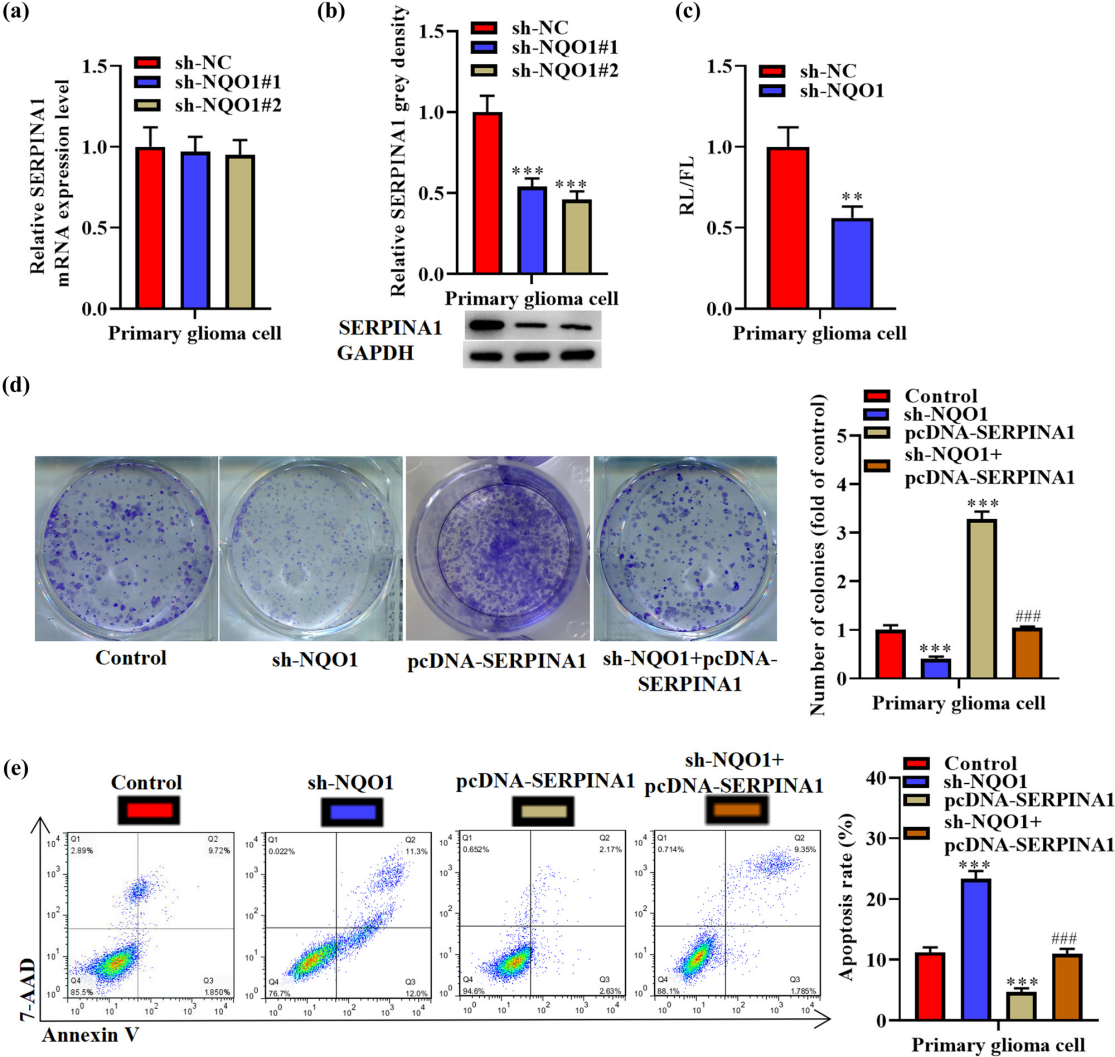
NQO1 binds to the 3′UTR of *SERPINA1* mRNA and suppresses its translation, and *SERPINA1* rescues effects of sh-NQO1 on proliferation and apoptosis of primary glioma cells. (a) Expression of *SERPINA1* in sh-NQO1#1/2 transfected primary glioma cells was assessed by qRT-PCR. One-way ANOVA was conducted. (b) *SERPINA1* protein levels in sh-NQO1#1/2 transfected primary glioma cells. One-way ANOVA was conducted. (c) The ratios of FL/RL activity in sh-*NQO1* transfected primary glioma cells. Student’s *t* test was conducted. (d) Colony formation assay results and quantitative analysis of primary glioma cells after transfection with sh-NQO1, pcDNA-SERPINA1, sh-NQO1 + pcDNA-SERPINA1. One-way ANOVA was conducted. (e) Annexin V/7AAD double staining assay results and quantitative analysis of primary glioma cells after indicated transfections. One-way ANOVA was conducted. Data are expressed as means ± SEM (**p < 0.01, ***p < 0.001 vs sh-NC or control, ^###^ *p* < 0.001 vs sh-NQO1, *n* = 3).

sh-NQO1 did not affect *SERPINA1* mRNA (Figure 5a) but decreased its protein expression in primary glioma cells (Figure 5b). Silencing of *NQO1* reduced the luciferase activity of psiCHECK2-SERPINA1-3′UTR in primary glioma cells (Figure 5c). Moreover, cell proliferation and apoptosis assays were conducted using primary glioma cells. The results revealed that sh-NQO1 reduced colony number and increased cell apoptosis rate, while pcDNA-SERPINA1 had inverse effects. pcDNA-SERPINA1 rescued the effects of sh-NQO1 in the primary glioma cell proliferation and apoptosis (Figure 5d–e).

### 3.6 miR-1321 is a negative regulator upstream *SERPINA1*

**Figure 6:**
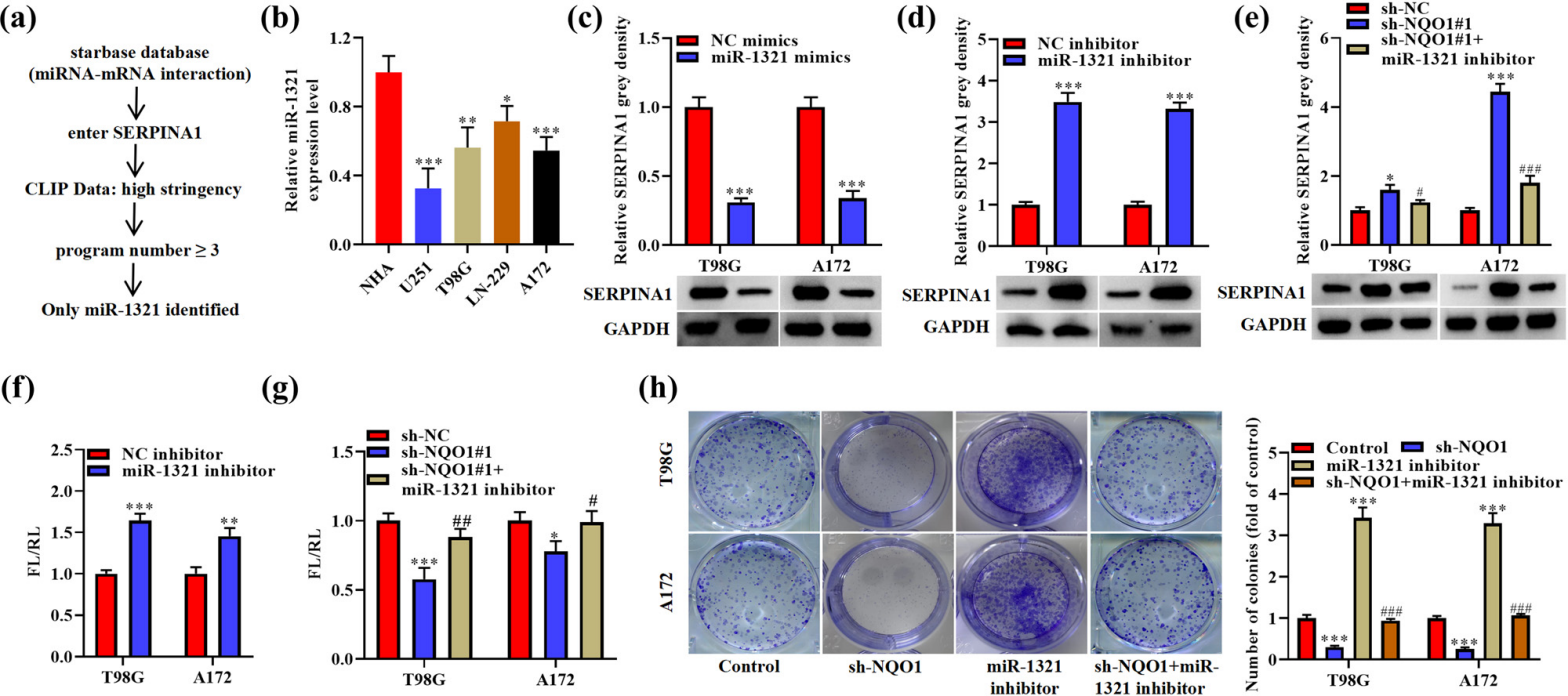
MiR-1321 is a negative regulator upstream *SERPINA1*. (a) A brief flow chart shows how miR-1321 was identified as a hit. (b) miR-1321 expression in NHA, U251, T98G, LN-229, and A172 cell lines. One-way ANOVA was conducted. (c and d) *SERPINA1* protein expression in T98G and A172 cells after transfection with miR-1321 mimics or inhibitor. Student’s *t* test was conducted. (e) *SERPINA1* protein levels in T98G and A172 cells after transfection with sh-NQO1#1 or sh-NQO1#1 + miR-1321 inhibitor. One-way ANOVA was conducted. (f) The ratios of FL/RL activity of psiCHECK2-SERPINA1-3′UTR in T98G and A172 cells after transfection with miR-1321 mimics. Student’s *t* test was conducted. (g) Relative luciferase activity of psiCHECK2-SERPINA1-3′UTR in T98G and A172 cells after transfection with sh-NQO1#1 or sh-NQO1#1 + miR-1321 inhibitor. One-way ANOVA was conducted. (h) Colony formation assay results and quantitative analysis of T98G and A172 cells after transfection with sh-NQO1#1 or sh-NQO1#1 + miR-1321 inhibitor. One-way ANOVA was conducted. Data are expressed as means ± SEM (*p < 0.05, **p < 0.01, ***p < 0.001 vs the first group in each panel, ^#^ *p* < 0.05, ^##^ *p* < 0.01, ^###^ *p* < 0.001 vs sh-NQO1, *n* = 3).

Using the starBase online database and under the condition of high stringency of CLIP Data and program number ≥3, miR-1321 was identified to potentially target *SERPINA1* (Figure 6a). miR-1321 shows downregulated expression in glioma cells than control NHA cell line (Figure 6b). miR-1321 mimics decreased *SERPINA1* protein expression, while miR-1321 inhibitor caused the upregulation of *SERPINA1* protein expression (Figure 6c–d) and rescued the suppressive effect of sh-NQO1 in *SERPINA1* protein expression (Figure 6e). miR-1321 inhibitor increased the expression of psiCHECK2-SERPINA1-3′UTR (Figure 6f) and rescued the inhibitory effect of sh-NQO1 on psiCHECK2-SERPINA1-3′UTR (Figure 6g). Moreover, miR-1321 inhibitor increased colony number of T98G and A172 cells and rescued the effects of sh-NQO1 in cell proliferation (Figure 6h).

## 4 Discussion

In the present work, we confirmed that *NQO1* functions as an RNA-binding protein (RBP) that binds with *SERPINA1* mRNA and enhances its translation, facilitating the proliferation of glioma cells by reducing the apoptosis. *NQO1* binds with *SERPINA1* mRNA and significantly increases its translation, thus boosting glioma tumorigenesis. Moreover, miR-1321 targets *SERPINA1* mRNA and represses its translation. MiR-1321 exerts an opposite effect of *SERPINA1.* The mutual effect of miR-1321 and *NQO1* on *SERPINA1* will cast new direction for treatment of glioma in the future exploration.

Glioma has been known as a primary malignant carcinoma in central systema nervosum [22]. Many studies about malignant cancers focused only on gene coding proteins, and their interactions are in lack of exploration [23]. Alpha-1 antitrypsin, the protein encoded by *SERPINA1*, is synthesized mainly by pulmonary alveolar cells, macrophages, and hepatocytes and modulates protease and corresponding inhibitors to defend glioma cells from host offense [24]. It also inhibits the cytotoxic reactions of lymphocytes [25,26,27,28]. The high expression of *SERPINA1* may protect tumor cells from enzymes and immune system [29]. We found that high expression of *SERPINA1* marks poor overall survival in glioma. The results of function assays showed that *SERPINA1* increased glioma cell proliferation by suppressing apoptosis.

*NQO1* binds with many mRNAs including *SERPINA1* [30,31]. We used shRNAs against *NQO1* to analyze the effect of *NQO1* silence on *SERPINA1*, which showed the same results as previous work that it downregulated the expression of *SERPINA1* at the protein level but not the mRNA level [30,31]. Through the luciferase reporter assay, our findings suggested that *NQO1* binds to the 3′UTR of *SERPINA1* mRNA. Considering the pro-proliferative and anti-apoptotic effects of *SERPINA1* in glioma, we had the hypothesis that by upregulating the expression of *SERPINA1*, *NQO1* would enhance the proliferation of glioma cells and suppress apoptosis, and such hypothesis was confirmed by the rescue assays.

*NQO1* may compete with the RBP suppressing *SERPINA1* translation or cooperate with RBP promoting *SERPINA1* translation. It is also possible that *NQO1* competes with certain microRNA (miRNA) that suppresses *SERPINA1* translation. In this study, we identified miR-1321 as a negative regulator upstream *SERPINA1*. MiR-1321 bond with *SERPINA1* 3′UTR and reduced its protein expression. miR-1321 inhibitor rescued the negative effect of sh-NQO1 on psiCHECK2-SERPINA1-3′UTR activity and *SERPINA1* protein expression. Moreover, miR-1321 shows downregulation in glioma cells and its silencing reduced glioma cell proliferation, indicating the tumor suppresser role of miR-1321 in glioma.

What are known until now based on abovementioned studies include: (1) *SERPINA1* is expressed in human glioma tissues; (2) NQO1 binds with *SERPINA1* 3′UTR in human hepatoma HepG2; and (3) NQO1 increases the proliferation of glioma cell lines. The novel findings of this study are listed as follows: (1) demonstrating the pro-proliferative and anti-apoptotic roles of *SERPINA1* in both glioma cell lines and primary glioma cells as well as the same function of *NQO1* in primary glioma cells; (2) confirming the binding of *NQO1* and *SERPINA1* 3′UTR in glioma cells on the basis of a previous study showing their binding in another cancer cell line; (3) revealing that miR-1321 binds to *SERPINA1* 3′UTR to suppress *SERPINA1* translation, indicating that *NQO1* may compete with miR-1321 to enhance *SERPINA1* expression; and (4) showing the antiproliferative function of miR-1321 in glioma cells.

In conclusion, our work demonstrates that in glioma cells, *NQO1* enhances the translation of *SERPINA1* mRNA by binding with 3′UTR and thus promotes the function of *SERPINA1* by suppressing apoptosis and enhancing the proliferation. Moreover, miR-1321 is a negative regulator of *SERPINA1*. *NQO1* may compete with miR-1321 to bind with *SERPINA1* 3′UTR and thus enhances its translation. This study suggested that *NQO1* may have similar effects on other target mRNAs and brings novel solution of antitumor treatments for glioma in the future work.

## Abbreviations

*SERPINA1*
: serpin family A member 1
*NQO1*
: nicotinamide adenine dinucleotide phosphate quinone oxidoreductase
mRNA: messenger RNA
shRNA: short hairpin RNA
SEM: standard error of the mean
GBM: glioblastoma multiforme
cDNA: complementary DNA
DAPI: 4′,6-diamidino-2-phenylindole
RBP: RNA-binding protein
RL: Renilla luciferase
FL: Firefly luciferase
